# Clinical Outcomes and Challenges in the Management of Spondylodiscitis in Patients With Intravenous Drug Abuse: A Multicenter Retrospective Study

**DOI:** 10.1177/21925682251399092

**Published:** 2025-11-18

**Authors:** Carolin Albrecht, Max Delank, Maria Wostrack, Claudius Jelgersma, Dimitri Tkatschenko, Julia Onken, Jonathan Neuhoff, Peter Vajkoczy, Bernhard Meyer, Ann-Kathrin Joerger

**Affiliations:** 1 Department of Neurosurgery, TUM University Hospital Rechts der Isar, 9184 Technical University, Munich, Germany; 2 Department of Neurosurgery, Berlin Institute of Health, 14903 Charité University Hospital, Berlin, Germany; 3 Center for Spinal Surgery and Neurotraumatology, RinggoldDI: 72067 Berufsgenossenschaftliche Unfallklinik Frankfurt am Main, Frankfurt am Main, Germany

**Keywords:** spondylodiscitis, spinal infection, intravenous drug abuse, people who inject drugs, vertebral osteomyelitis

## Abstract

**Study Design:**

Retrospective multicenter study.

**Objective:**

To examine the epidemiology and clinical outcomes of spondylodiscitis in patients with intravenous drug abuse (IVDU) and compare them with non-IVDU patients.

**Methods:**

Data from 575 patients diagnosed with spondylodiscitis between 1 January 2018 and 31 December 2023 from three high-volume spine centers was analyzed. Of these, 33 (5.74%) were patients with IVDU, and 542 (94.26%) were non-IVDU patients. Clinical characteristics, bacterial spectrum, and treatment outcomes, including revision surgery rates and cure rates, were compared.

**Results:**

Patients with IVDU were significantly younger (mean age 43.9 ± 9.1 years) compared to non-IVDU patients (mean age 70.5 ± 11.9 years) (*P* < .0001). The median Charlson Comorbidity Index (CCI) was significantly lower in IVDU patients (1, IQR: 0-3) compared to non-IVDU patients (4, IQR: 3-6) (*P* < .0001). The bacterial spectrum was similar between both groups, with *Staphylococcus aureus* as the most frequent pathogen. Revision surgery rates were comparable; however, among patients requiring revision, recurrent or progressive discitis was more frequently the cause in IVDU patients (55.6%) compared to non-IVDU patients (17.9%). At 10-week follow-up, 87.9% of non-IVDU patients were cured, while only 57.9% of IVDU patients achieved a cure (*P* = .0018).

**Conclusions:**

IVDU patients with spondylodiscitis are younger and have fewer comorbidities than non-IVDU patients. Contrary to common assumptions, they do not present with more severe infections. However, they experience higher relapse and progression rates, highlighting the need for tailored treatment strategies in this high-risk group.

## Introduction

Spondylodiscitis is on the rise in Europe and the United States, contributing to a significant socioeconomic burden.^1-4^ In Germany, for example, the incidence of spondylodiscitis increased by 104% from 5.4 to 11.0 cases per 100 000 individuals between 2005 and 2021.^4^ This trend can be attributed to several factors, including an aging population and a growing number of multimorbid and immunocompromised individuals.^5-7^ Additionally, advancements in diagnostic techniques and broader access to diagnostic modalities have improved early detection of cases.^8^

Despite the establishment of diagnostic and therapeutic guidelines, the treatment of spondylodiscitis remains non-standardized and varies across healthcare facilities. Known risk factors for spondylodiscitis include advanced age, immunosuppression (eg, due to medication or HIV infection), diabetes, alcoholism, malnutrition, and malignancy.^9^

Among these, patients with intravenous drug abuse (IVDU) represent a particularly vulnerable and challenging subgroup. They not only frequently exhibit comorbidities associated with the aforementioned risk factors but also engage in behaviors, such as the recurrent use of unsterile needles, that significantly increase their risk of soft tissue abscesses, bloodstream infections, and sepsis.^9^ The most common admission diagnoses in patients with IVDU are bacteremia, abscesses, endocarditis, cellulitis, sepsis, and osteomyelitis.^10^ The management of spondylodiscitis in patients with IVDU is further complicated by issues related to treatment adherence. Effective treatment of spondylodiscitis typically requires a prolonged course of intravenous followed by oral antibiotic therapy—commonly lasting up to 12 weeks at our center—which demands a high level of patient compliance. However, studies have shown that compliance in patients with IVDU is often suboptimal. For example, research from Vancouver found that up to 30% of patients with IVDU discharged themselves against medical advice during hospitalization.^11^

In Germany, the prevalence of IVDU is lower than in many regions globally and has remained relatively stable over the last few decades. According to data from the United Nations Office on Drugs and Crime (UNODC),^12^ the prevalence of heroin use in Germany in 2021 was 0.20%, compared to 1.12% in the United States in 2020. Globally, the number of people who inject drugs was estimated to be approximately 13.2 million, as reported in the UNODC World Drug Report 2023.^13^

Because of this comparably low prevalence, literature on the treatment and outcomes of spondylodiscitis patients with IVDU in Germany is scarce. This study aims to address this gap by examining the epidemiology and clinical course of spondylodiscitis in this distinct patient population in Germany, providing insights into the unique challenges and outcomes associated with their care.

## Methods

### Population

We conducted a retrospective multicenter study involving three comparable Level 1 high-volume spine centers in metropolitan areas across three different regions of Germany, which have been noted for higher rates of IVDU. Patients with spondylodiscitis and IVDU treated at these centers were included between 1 January 2018 and 31 December 2023. The study evaluated demographic parameters, disease-specific parameters, and disease outcomes. These findings were compared with a cohort of consecutive patients with spondylodiscitis without IVDU treated at one of the three centers during the same period.

### Diagnostics and Treatment

All patients underwent whole spinal magnetic resonance imaging (MRI) examinations. Computed tomography (CT) scans were performed at a minimum in the area of the affected segments to assess bony destruction or as an alternative in cases where MRI was contraindicated. In such cases, CT imaging was often supplemented with fluorodeoxyglucose positron emission tomography (FDG-PET).

Surgical therapy was performed in nearly all cases, except in patients with significantly elevated perioperative risks, those who succumbed before surgery could be undertaken, or those who declined surgical intervention.

Radiographic instability parameters in the CT-scan, such as posterior wall involvement, vertebral body collapse greater than 50%, or segmental kyphosis, were considered during surgical planning but were not absolute criteria for the indication of surgery itself. These factors specifically influenced the decision as to whether the affected vertebral body was included in the instrumentation. If not, vertebral body replacement was performed. In cases where the affected vertebral body was omitted, stabilization for spondylodiscitis of the thoracic or lumbar spine involved instrumentation two levels above and two below the affected segment, along with vertebral body replacement, usually performed in a second-stage procedure via a retroperitoneal or transthoracic approach. Whenever possible, and in the absence of neurological deficits, dorsal decompression was omitted, and percutaneous instrumentation was preferred to minimize blood loss, reduce surgical trauma, and shorten operative time, given that many patients were critically ill and often presented with impaired coagulation. If the vertebral body adjacent to the discitis was not severely destructed, it was included in the instrumentation, and the construct typically spanned two levels above and two below the infected disc space. A second-stage procedure was then performed to debride the intervertebral disc space via a retroperitoneal or transthoracic approach, with or without decompression depending on clinical symptoms, followed by fusion using either a cage or autologous bone graft in combination with an antibiotic-loaded sponge. In cases of cervical spondylodiscitis without the aforementioned radiographic instability criteria, an anterior cervical discectomy and fusion (ACDF) with anterior plating was performed. In cases meeting instability criteria, posterior instrumentation was carried out, followed by anterior vertebral body replacement. If no neurological deficit was present, patients typically underwent percutaneous instrumentation without decompression. In contrast, in cases of neurological impairment and imaging evidence of compressive spinal empyema, decompression was performed—preferably via interlaminar fenestration in addition to percutaneous instrumentation, provided that the extent of the empyema made this approach feasible; otherwise, an extended laminectomy was performed as part of an open stabilization procedure.

Empirical antibiotic therapy was initiated promptly following biopsy collection or, in critically ill patients with elevated inflammatory markers, after obtaining blood cultures. The antibiotic regimen was subsequently adjusted based on the identified pathogen spectrum. The general recommendation regarding the duration of antibiotic therapy was at least two weeks intravenously, followed by 10 weeks orally. A longer duration was required, depending on the identified pathogen and the individual case.

Follow-up appointments were scheduled depending on the duration of antibiotic therapy, but most commonly took place shortly before discontinuation of oral antibiotics, typically 10 weeks after discharge. Cure was defined as the absence of neurological deficits, normalization of inflammatory markers (C-reactive protein = CRP, and leukocyte count), and resolution or significant improvement of pain and functional capacity. Routine imaging was only performed in cases of new or persistent symptoms.

### Statistics

For parameters with a confirmed normal distribution, Welch’s *t*-test (*t*-test for unequal variances) was applied to compare metric variables. In cases where normal distribution was not confirmed, the Mann-Whitney U test was utilized for analysis. Fisher’s exact test and Chi-square test were used to measure categorical variables. A *P*-value of <.05 was considered statistically significant.

Statistical analysis was performed with GraphPad Prism 10.3.1 (San Diego, California, USA).

### Declaration of Generative AI and AI-Assisted Technologies in the Writing Process

While preparing this work, the authors used ChatGPT3.5 (OpenAI, San Francisco, California, USA) and Grammarly (Grammarly, Inc, San Francisco, California, USA) to check spelling, grammar, and style. After using this service, the authors reviewed and edited the content as needed and took full responsibility for the publication’s content.

## Results

### Demographic Background

Over the 6-year study period, 575 patients with spondylodiscitis were included. Of these, 33 (5.74%) were intravenous drug users, and 542 (94.26%) were non-intravenous drug users. 21 of the 33 (63.6%) IVDU patients reported active drug abuse. The male-to-female ratio was 1.95:1 for non-intravenous drug users and 1.20:1 for intravenous drug users (*P* = .1900) (Table 1). Patients with IVDU were significantly younger (mean age 43.9 (+/− 9.1) years) compared to patients without IVDU (mean age 70.5 (+/− 11.9) years) (*P* < .0001) (Table 1). Median body mass index (BMI) was significantly lower for patients with IVDU (23.1 kg/m^2^, IQR: 20.7 – 26.8 kg/m^2^) compared to patients without IVDU (25.8 kg/m^2^, IQR: 23.0 – 29.9 kg/m^2^) (*P* = .0013) (Table 1). Median Charlson Comorbidity Index (CCI) was significantly lower for patients with IVDU (1, IQR: 0 – 3) compared to patients without IVDU (4, IQR: 3 – 6) (*P* < .0001) (Table 1).

**Table 1.** Patient Demographics

Parameter	Non-IVDU (*n* = 542)	IVDU (*n* = 33)	*P*
Age (y)	70.5 ± 11.9	43.9 ± 9.1	**<.0001**
Male-female-ratio	1.95:1	1.20:1	.190
BMI (kg/m2)	25.8 (23-29.9)	23.1 (20.7-26.8)	**.0013**
ASA	3 (2-3)	3 (2-3)	.9505
CCI	4 (3-6)	1 (0-3)	**<.0001**
Active substance use	n.a.	21 (63.6%)	

IVDU = intravenous drug abuse, BMI = Body Mass Index, CCI = Charlson Comorbidity Index, ASA = American Society of Anesthesiologists. To compare age Welch’s t-test was used. To compare sex Fisher’s exact text was used. To compare BMI, ASA and CCI Mann-Whitney U test was used. A p-value of < 0.05 was considered statistically significant.

None of the patients with IVDU had a concurrent HIV infection, while 13 (39.4%) had active hepatitis, 6 (18.2%) had chronic hepatitis, and 6 (18.2%) had a history of resolved hepatitis.

### Clinical Presentation and Infection Characteristics

**Figure 1.**
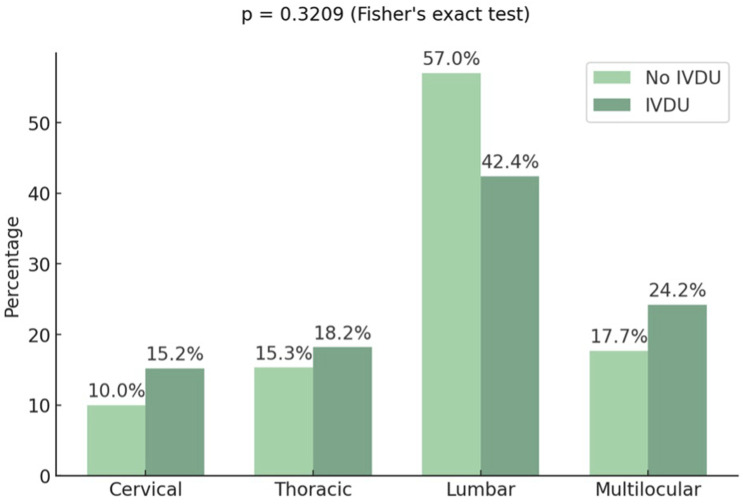
Distribution of Affected Spinal Segments. To Compare Patients With and Without IVDU Fisher’s Exact Test was Used. A *P*-value of < .05 was Considered Statistically Significant

The lumbar spine was the most commonly affected region in both groups, followed by the thoracic spine (Figure 1). There was no significant difference between the two groups regarding the presence of a paraspinal abscess or epidural empyema (*P* = .0970 and *P* = .1541, respectively) (Table 2).

**Table 2.** Clinical and Laboratory Findings

Parameter	Non-IVDU (*n* = 542)	IVDU (*n* = 33)	*P*
Paraspinal abscess	38.4%	54.5%	.0970
Epidural empyema	44.6%	57.6%	.1541
CRP (mg/dL, cutoff 0.5) (IQR)	7.8 (3.2-17.3)	9 (2.7-14.4)	.7534
Leukocytes (G/L, cutoff 10) (IQR)	9.3 (7.1-12.4)	9.9 (7.5-13.2)	.4068
Motor deficits at admission	36%	36.4%	>.999
Bladder/Bowel dysfunction at admission	13.7%	12.1%	>.999
Presumed infection focus (most frequent)			
Urogenital	10.3%	0%	n.a.
Joint empyema	9.4%	15.2%	
Odontogenic	7.2%	6.1%	
Unknown	27.5%	63.6%	
Endocarditis	5.0%	3.1%	>.999
Prior spinal surgery as site of infection	147 (27.1%)	2 (6.1%)	n.a.
Early postoperative infection	19	0	
Delayed postoperative infection	110	2	
Recurrent spondylodiscitis	15	0	
Infection per continuitatem	3	0	

CRP = C-reactive protein. To compare CRP and Leukocyte levels Mann-Whitney U test was used. To compare the rate of abscess, empyema, bladder/bowel dysfunction, and endocarditis, Fisher’s exact test was used. A p-value of < 0.05 was considered statistically significant.

The median CRP level at diagnosis was elevated in both groups, measuring 9 mg/dL (IQR: 2.7-14.4 mg/dL) in the IVDU group and 7 mg/dL (IQR: 3.2-17.3 mg/dL) in the non-IVDU group, compared to the cutoff value of 0.5 mg/dL (Table 2). However, no significant difference was observed between the groups (*P* = .7534). In contrast, the median leukocyte count at diagnosis was not elevated and remained similar across both groups relative to the cutoff value of 10 G/L (Table 2).

36.4% of IVDU patients presented with paresis at admission, similar to the 36.0% of non-IVDU patients (*P* > .9999) (Table 2). Additionally, the rate of bladder or bowel dysfunction at admission was comparable between groups (*P* > .999) (Table 2). Among IVDU patients, there was no significant difference in neurological status or clinical condition between those treated surgically and those managed conservatively. In contrast, among non-IVDU patients, a significantly higher proportion of those treated conservatively were septic at admission, whereas they presented with neurological deficits less frequently at both admission and discharge (supplemental table).

The most frequently presumed source of infection in patients with IVDU was hematogenous spread from peripheral infectious foci, including extremity abscesses or empyema in peripheral joints (15.2%) and odontogenic infections (6.1%) (Table 2). Infective endocarditis was identified in 3.1% of patients with IVDU. In 63.6% of these patients, no definitive infectious focus could be determined. Among patients without a history of IVDU, the most frequently presumed sources of infection were urogenital infections (10.3%) and extremity abscesses or joint empyema (9.4%) (Table 2). Endocarditis was present in 5.0% of non-IVDU patients (*P* > .999). No infectious focus could be identified in 27.5% of these cases. In both groups, the presumed mechanism of infection was hematogenous dissemination from the respective primary focus.

To assess the role of previous spinal surgery as the site of infection, we analyzed the frequency and timing of prior interventions at the same spinal level as the current infection. In the IVDU group, only two patients (6.1%) had a history of prior spinal surgery at the same spinal level, performed four and five years prior to the current presentation, respectively. Given the long interval, a postoperative origin is unlikely, and these cases are more consistent with hematogenous spread. In contrast to the IVDU patients, in 27.1% of non-IVDU patients, a prior surgical procedure at the same spinal level was identified as the site of infection. Of the 147 patients included, 19 developed early postoperative infections within 30 days of spinal surgery at the same level. These consisted of 10 surgical site infections and 9 cerebrospinal fluid leaks. In 15 patients, recurrent spondylodiscitis was diagnosed. This referred to a re-infection at the same spinal level previously operated on due to spondylodiscitis, confirmed by imaging, clinical symptoms, and re-elevated inflammatory markers.

The remaining 110 patients presented with delayed infections, which occurred more than 30 days postoperatively, after complete wound healing. These infections developed at the site of previous spinal surgery performed for other indications than spondylodiscitis, such as decompression or instrumentation for degenerative spine disease or fractures. In three cases, the infection was attributed to direct contiguous spread *(per continuitatem*) from adjacent surgical sites, facilitated by fistula formation following ENT surgery (cervical spine), gynecological surgery (lumbar spine), or carotid surgery (cervical spine).

### Microbiological Findings

**Figure 2.**
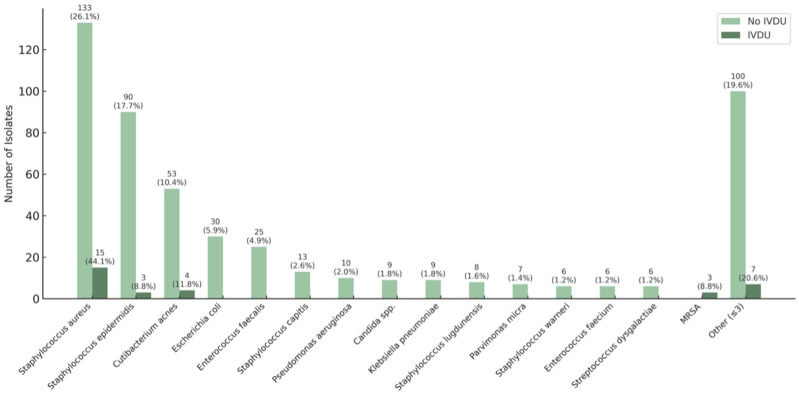
Bacterial Spectrum of Non-IVDU and IVDU Patients. Note: Multiple Pathogens Could be Isolated Per Patient; Percentages may Therefore Exceed 100%. MRSA = Methicillin-Resistant Staphylococcus Aureus

Pathogen detection was successful in 81.8% of cases among patients with IVDU and in 77.5% of cases of non-intravenous drug users (*P* = .6703) (Table 3). In patients without IVDU, a total of 509 pathogens were detected. The three most common pathogens were *Staphylococcus aureus* (133, 26.13%), *Staphylococcus epidermidis* (90, 17.68%), and *Cutibacterium acnes* (53, 10.41%) (Figure 2). In patients with IVDU, a total of 34 pathogens were identified. Here as well, the most common pathogens were *Staphylococcus aureus* (15, 44.12%), *Cutibacterium acnes* (5, 11.76%), and *Staphylococcus epidermidis* (3, 8.82%) (Figure 2).

**Table 3.** Microbiological Findings

Parameter	Non-IVDU (*n* = 542)	IVDU (*n* = 33)	*P*
Positive pathogen detection	77.5%	81.8%	.6703
Total no. of pathogens	509	34	
Staphylococcus aureus	133 (26.13%)	15 (44.12%)	
Staphylococcus epidermidis	90 (17.68%)	5 (11.76%)	
Cutibacterium acnes	53 (10.41%)	3 (8.82%)	
Escherichia coli	30 (5.89%)	0	
Enterococcus faecalis	25 (4.91%)	0	
Staphylococcus capitis	13 (2.55%)	0	
Pseudomonas aeruginosa	10 (1.96%)	0	
Candida spp.	9 (1.77%)	0	
Klebsiella pneumoniae	9 (1.77%)	0	
Staphylococcus lugdunensis	8 (1.57%)	0	
Parvimonas micra	7 (1.38%)	0	
Staphylococcus warneri	6 (1.12%)	0	
Enterococcus faecium	6 (1.12%)	0	
Streptococcus dysgalactiae	6 (1.12%)	0	
MRSA	0	3 (8.82%)	

To compare positive pathogen detection rate Fisher’s exact test was used. A p-value of < 0.05 was considered statistically significant.

### Treatment and Follow-Up

Of the patients with IVDU, 32 (97.0%) underwent surgical treatment, compared to 522 (96.3%) of the patients without IVDU (*P* > .9999) (Table 4). The rate of revision surgeries was comparable between the two groups (27.3% with IVDU, 25.8% without IVDU, *P* = .8394) (Table 4). The most common reasons for revision surgery in patients with IVDU were recurrent or progressive discitis (5, 55.6%), wound healing disorders (2, 22.2%), screw loosening (1, 11.1%), and instability (1, 11.1%). In contrast, the most common reasons in patients without IVDU were wound healing disorders (72, 51.4%), recurrent or progressive discitis (25, 17.9%), and postoperative bleeding (11, 7.9%) (*P* = .1229) (Table 4). The proportion of patients with paresis or bladder/bowel dysfunction at discharge did not differ between the two groups (*P* = .3416 and *P* = .2926, respectively) (Table 5). A follow-up of at least 10 weeks after discharge was available for 57.6% (19) of patients with IVDU, while 42.4% (14) were lost to follow-up. Among patients without IVDU, a follow-up of at least 10 weeks was available for 51.7% (280), 40.2% (218) were lost to follow-up, and 8.1% (44) had died earlier (*P* = .2316) (Table 5). Among the patients with a 10-week follow-up, a more significant proportion of patients without IVDU were cured of spondylodiscitis (87.9%) compared to patients with IVDU, whose cure rate was only 57.9% (*P* = .0018) (Table 5).

**Table 4.** Treatment Characteristics

Parameter	Non-IVDU (*n* = 542)	IVDU (*n* = 33)	*P*
Treatment			
Surgical	73.5%	97%	>.999
Conservative	26.5%	3%	
Revision	25.8%	27.3%	.8394
Reason for revision			
Wound healing	51.4%	22.2%	.1229
Screw loosening	10.7%	11.1%	
Instability	2.9%	11.1%	
Hematoma	7.9%	0%	
Progressive discitis	17.9%	55.6%	

To compare treatment strategy and rate and type of revision surgery Fisher’s exact test was used. A p-value of < 0.05 was considered statistically significant.

**Table 5.** Clinical Outcome Parameters

Parameter	Non-IVDU (*n* = 542)	IVDU (*n* = 33)	*P*
Symptoms at discharge			
Paresis	33.1%	24.2%	.3416
Bladder/Bowel dysfunction	13.7%	12.1%	.2926
90-day mortality	8.1%	0%	.2316
10 weeks follow-up	280 (51.7% of total n)	19 (57.6% of total n)	.2316
- Cured	246 (87.9% of 280)	11 (57.9% of 19)	**.0018**

To compare rates of paresis and bladder/bowel dysfunction Fisher’s exact test was used. To compare 10 weeks follow-up and 90-day mortality rates Chi square test was used. A p-value of < 0.05 was considered statistically significant.

## Discussion

This study examined the epidemiology and clinical course of spondylodiscitis patients with IVDU.

### Demographic Background

Age is an independent risk factor for spondylodiscitis,^14^ with peak incidence in Germany among those aged 70-89 years.^4^ In this study, the mean age of non-IVDU patients was 70.5 years, while IVDU patients were approximately 25 years younger. Furthermore, patients with IVDU had fewer comorbidities, as indicated by a significantly lower CCI than those without IVDU. This finding was expected and is consistent with previous reports.^9,15-18^ Consistent with previous studies reporting a strong male predominance among IVDU patients,^9,15-17^ our cohort showed a higher proportion of male patients in the IVDU group as well; however, this difference was not statistically significant compared to non-IVDU patients.

Interestingly, none of the patients with IVDU in our study had a concurrent HIV infection, despite the reported HIV prevalence of 4.9% among people who inject drugs in Germany.^13^ In contrast, approximately 58% of the IVDU patients had either active or chronic hepatitis, a rate consistent with the 65.5% prevalence of hepatitis C infections among this population in Germany.^13^

### Clinical Presentation and Infection Characteristics

Data regarding the most commonly affected spinal region in patients with IVDU differ across studies, varying between cervical^16,19^ and lumbar.^9,15,17^ In our study, the lumbar spine was the most frequently affected region in patients with and without IVDU. The presence of an epidural abscess in addition to spondylodiscitis was higher in our study than in the literature.^9,17^ However, the rate of patients presenting with neurological deficits was approximately one-third in both groups, consistent with previous figures.^20^ In our study, the mean CRP level was elevated in both groups, while the mean leukocyte count was not. This supports literature findings, which show CRP has higher sensitivity than leukocyte count in diagnosing spondylodiscitis.^21^

In our study, the vast majority of patients underwent surgical treatment. Among IVDU patients, there was no significant difference in neurological status or clinical condition between those treated surgically and those managed conservatively. This finding was expected, as the conservative group in this subgroup included only one patient.

In contrast, among non-IVDU patients, a significantly higher proportion of those treated conservatively were septic at admission, while neurological deficits were less common in this group both at admission and at discharge. This, too, was anticipated, as the decision to pursue conservative management in these cases was primarily based on the patients’ critical condition—severe sepsis with pronounced cardiopulmonary instability rendered them inoperable.

Regarding the source of infection, it is not always possible to differentiate between the initial focus of infection and a concomitant infection. Among patients with IVDU, in whom an infectious focus was identified, the most frequent infectious foci were extremity abscesses and joint empyemas, which was expected as these include abscesses caused by needle injections. In general, abscesses are the second most common admission diagnosis among people who inject drugs.^10^ Interestingly, the rate of patients with concomitant endocarditis in both groups was notably lower than what has been previously reported, where rates of up to 27% have been described.^22^

Contrary to the commonly held assumption that patients with IVDU present with more severe forms of spondylodiscitis, our data showed no significant differences in the severity of infection between IVDU and non-IVDU patients, as reflected by similar rates of neurological deficits, elevated inflammatory markers, and concomitant endocarditis.

### Microbiological Findings

The pathogen detection rates were 81.8% for patients with IVDU and 77.5% for patients without IVDU. These rates are consistent with those reported in previous studies for patients with IVDU^9^ and without.^23^ The bacterial spectrum did not differ between the two groups, with *Staphylococcus aureus* being the most frequently detected bacterium in both groups. For spondylodiscitis in general, *Staphylococcus aureus* is known to be the most frequent causative pathogen in Europe.^24^ Recent studies have also found *Staphylococcus aureus* to be the most frequently detected bacterium in spondylodiscitis patients with IVDU.^9,16,17,25^ However, less common bacteria such as *Serratia marcescens* have also been described to be prevalent in people who inject drugs, causing osteomyelitis.^26,27^ Especially in the 1970s, *Pseudomonas aeruginosa* was the most frequent pathogen causing spondylodiscitis in IVDU patients.^19,28^ In our study, *Serratia marcescens* and *Pseudomonas aeruginosa* were not detected at all in the group of IVDU patients. This can be explained by changes in drug preparation practices. In the 1970s and early 1980s, the drug pentazocine-tripelenamine, which is soluble in cold water, was commonly abused.^29^ Drug preparation methods included dissolving the crushed tablet in tap or toilet water, often serving as a reservoir for *Pseudomonas aeruginosa*. In contrast, during the later 1980s, heroin became more widespread, and its preparation required heating, which likely reduced bacterial contamination.

### Treatment and Follow-Up

As per standard practice in the participating spine centers, nearly all patients in both groups underwent surgical treatment for spondylodiscitis. Interestingly, the two groups’ absolute revision rate was comparable and slightly higher than the 19% reported in a study from a comparable center in Hamburg, Germany.^30^ This higher rate in our study may be explained by the fact that the Hamburg study reported immediate short-term complications requiring surgical revision. In contrast, our study also included long-term complications, such as implant failure or recurrence of spondylodiscitis, that required surgical intervention. A critical finding of our study was that the reasons for revision differed between patients with and without IVDU. Patients with IVDU were substantially more likely to experience a relapse or progressive spondylodiscitis compared to those without IVDU. This was also reflected in the healing rate after a 10-week follow-up, which was significantly lower in patients with IVDU. There are only a few studies on patients with IVDU and spondylodiscitis, and treatment and follow-up rates vary widely among them, so our results are difficult to compare with previous data. A study in which patients received the most comparable treatment to our study was conducted by Wang et al^16^ In their study, 86% of patients with IVDU underwent surgical treatment. The authors reported an 18% early hardware failure rate for patients with IVDU, but since the follow-up rate for IVDU patients was 0%, they did not provide any data on long-term revision rates or relapse rates. Considering that patients with IVDU are generally regarded as non-compliant, the follow-up rate of almost 58% observed in our study was notably high for this particular patient population. However, since our data are retrospective, they do not allow for a reliable assessment of whether patients with IVDU consistently adhered to their antibiotic treatment. An important factor that must be taken into account.

## Limitations and Strengths

This study is retrospective, which introduces the potential for recall bias and incomplete data. Both groups exhibited high loss-to-follow-up rates; however, the rate in the IVDU group was expected to be even higher. A large-scale prospective trial with standardized follow-up would be necessary to address this limitation; however, this would be difficult to realize given the non-compliant nature of the special population of intravenous drug users.

Another potential limitation of this study is the sample size of 33 patients, which may initially seem small and could limit the generalizability of the findings. However, this number must be considered in the context of the overall low prevalence of intravenous drug use in Germany, estimated at only 0.20%,^12^ with an even smaller proportion of affected individuals being sufficiently compliant to seek medical care. Strengthening the study’s significance is the fact that the data were collected from three metropolitan areas across Germany, which have been noted for higher rates of IVDU. Additionally, all three centers are high-volume spine centers, further supporting the reliability of the findings.

To our knowledge, this is the first study in Germany to analyze this specific subgroup of patients across three metropolitan areas, providing valuable insights into the epidemiology and clinical course of the disease.

## Conclusion

Patients with IVDU and spondylodiscitis were significantly younger, more often male, and had fewer comorbidities compared to non-IVDU patients. Although no cases of HIV co-infections were documented, the prevalence of hepatitis B or C was notably high in the IVDU group, reflecting the specific risk profile of this population. Contrary to commonly held assumptions, the severity of infection as indicated by neurological deficits at presentation, elevated inflammatory markers, concomitant endocarditis, and microbiological profiles did not differ significantly between IVDU and non-IVDU patients. Both groups showed similar distributions of pathogens, with *Staphylococcus aureus* being the predominant organism.

The presumed route of infection differed notably between groups: In IVDU patients, extremity abscesses and odontogenic infections represented the most frequent presumed sources of infection. In non-IVDU patients, presumed infectious foci were more often urogenital infections.

In the group of non-IVDU patients, a comparably high number had a prior surgical procedure at the same spinal segment identified as the site of infection. Notably, only a small proportion of these cases occurred in the early postoperative period, while the majority were delayed infections.

While the overall revision rates were comparable, recurrent or progressive discitis was more frequently the indication for revision in IVDU patients.

Surprisingly, IVDU patients demonstrated acceptable compliance in terms of follow-up attendance. However, at 10-week follow-up, the rate of successful infection control was significantly lower in IVDU patients despite comparable initial management strategies and similar rates of surgical intervention. This highlights not only the biological but also the psychosocial complexity in effectively treating these patients. Taken together, our findings show that although the clinical presentation of spondylodiscitis in IVDU patients may not be more severe per se, outcomes tend to be less favorable. This underscores the need for close clinical surveillance and tailored treatment strategies for this vulnerable population.

## Supplemental Material

Suppplemental Material - Clinical Outcomes and Challenges in the Management of Spondylodiscitis in Patients With Intravenous Drug Abuse: A Multicenter Retrospective Study Suppplemental Material for Clinical Outcomes and Challenges in the Management of Spondylodiscitis in Patients With Intravenous Drug Abuse: A Multicenter Retrospective Study by Carolin Albrecht, Max Delank, Maria Wostrack, Claudius Jelgersma, Dimitri Tkatschenko, Julia Onken, Jonathan Neuhoff, Peter Vajkoczy, Bernhard Meyer, Ann-Kathrin Joerger in Global Spine Journal

## Data Availability

The primary data supporting the conclusions of this study are available from the corresponding author upon reasonable request.*

## References

[1] Lang S Walter N Schindler M , et al. The epidemiology of spondylodiscitis in Germany: a descriptive report of incidence rates, pathogens, In-Hospital mortality, and hospital stays between 2010 and 2020. J Clin Med. 2023;12(10):3373. doi:10.3390/jcm12103373 37240479 PMC10219516

[2] Issa K Diebo BG Faloon M , et al. The epidemiology of vertebral osteomyelitis in the United States from 1998 to 2013. Clin Spine Surg. 2018;31(2):E102-E108. doi:10.1097/BSD.0000000000000597 29135608

[3] Conan Y Laurent E Belin Y , et al. Large increase of vertebral osteomyelitis in France: a 2010-2019 cross-sectional study. Epidemiol Infect. 2021;149:e227. doi:10.1017/S0950268821002181 34612186 PMC8569834

[4] Kramer A Thavarajasingam SG Neuhoff J , et al. Epidemiological trends of pyogenic spondylodiscitis in Germany: an EANS spine section study. Sci Rep. 2023;13(1):20225. doi:10.1038/s41598-023-47341-z 37980371 PMC10657388

[5] Kehrer M Pedersen C Jensen TG Lassen AT . Increasing incidence of pyogenic spondylodiscitis: a 14-year population-based study. J Infect. 2014;68(4):313-320. doi:10.1016/j.jinf.2013.11.011 24296494

[6] Herren C von der Hoeh NH Zwingenberger S , et al. Spondylodiscitis in geriatric patients: what are the issues? Glob Spine J. 2023;13(1_suppl):73S-84S. doi:10.1177/21925682221121300 PMC10177302 37084348

[7] Martinson ML Lapham J . Prevalence of immunosuppression among US adults. JAMA. 2024;331(10):880-882. doi:10.1001/jama.2023.28019 38358771 PMC10870224

[8] Kouijzer IJE Scheper H de Rooy JWJ , et al. The diagnostic value of (18)F-FDG-PET/CT and MRI in suspected vertebral osteomyelitis - a prospective study. Eur J Nucl Med Mol Imag. 2018;45(5):798-805. doi:10.1007/s00259-017-3912-0 PMC5978906 29256136

[9] Chuo CY Fu YC Lu YM , et al. Spinal infection in intravenous drug abusers. J Spinal Disord Tech. 2007;20(4):324-328. doi:10.1097/BSD.0b013e31802c144a 17538358

[10] Lim J Pavalagantharajah S Verschoor CP , et al. Infectious diseases, comorbidities and outcomes in hospitalized people who inject drugs (PWID). PLoS One. 2022;17(4):e0266663. doi:10.1371/journal.pone.0266663 35443003 PMC9020696

[11] Jafari S Joe R Elliot D Nagji A Hayden S Marsh DC . A community care model of intravenous antibiotic therapy for injection drug users with deep tissue infection for reduce leaving against medical advice. Int J Ment Health Addiction. 2015;13(1):49-58. doi:10.1007/s11469-014-9511-4 PMC4320270 25685126

[12] UNODC . UNODC research - data portal – drug use & treatment. 2024. Accessed 05/01/2025. https://dataunodc.un.org/dp-drug-use-prevalence

[13] The World Drug Report 2023. United Nations Office on Drugs and Crime (UNODC), Vienna, Austria, published online: https://www.unodc.org/unodc/en/data-and-analysis/world-drug-report-2023.html

[14] Sur A Tsang K Brown M Tzerakis N . Management of adult spontaneous spondylodiscitis and its rising incidence. Ann R Coll Surg Engl. 2015;97(6):451-455. doi:10.1308/rcsann.2015.0009 26274746 PMC5126242

[15] DiGiorgio AM Stein R Morrow KD Robichaux JM Crutcher CL Tender GC . The increasing frequency of intravenous drug abuse-associated spinal epidural abscesses: a case series. Neurosurg Focus. 2019;46(1):E4. doi:10.3171/2018.10.FOCUS18449 30611170

[16] Wang Z Lenehan B Itshayek E , et al. Primary pyogenic infection of the spine in intravenous drug users: a prospective observational study. Spine. 2012;37(8):685-692. doi:10.1097/BRS.0b013e31823b01b8 22037525

[17] Ziu M Dengler B Cordell D Bartanusz V . Diagnosis and management of primary pyogenic spinal infections in intravenous recreational drug users. Neurosurg Focus. 2014;37(2):E3. doi:10.3171/2014.6.FOCUS14148 25081963

[18] Bilolikar VK Gleason B Kripke L Merrill R Whitaker C Handal J . Risk factors associated with pyogenic spinal infections among intravenous drug users and nonusers. Adv Orthop. 2024;2024:9938159. doi:10.1155/2024/9938159 39105127 PMC11300094

[19] Sapico FL Montgomerie JZ . Vertebral osteomyelitis in intravenous drug abusers: report of three cases and review of the literature. Rev Infect Dis. 1980;2(2):196-206. doi:10.1093/clinids/2.2.196 6771865

[20] Mylona E Samarkos M Kakalou E Fanourgiakis P Skoutelis A . Pyogenic vertebral osteomyelitis: a systematic review of clinical characteristics. Semin Arthritis Rheum. 2009;39(1):10-17. doi:10.1016/j.semarthrit.2008.03.002 18550153

[21] Maus U Andereya S Gravius S Ohnsorge JA Miltner O Niedhart C . [Procalcitonin (PCT) as diagnostic tool for the monitoring of spondylodiscitis]. Z für Orthop Unfallchirurgie. 2009;147(1):59-64. doi:10.1055/s-2008-1038974. Procalcitonin (PCT) als Verlaufsparameter der Spondylodiszitis. 19263315

[22] Koslow M Kuperstein R Eshed I Perelman M Maor E Sidi Y . The unique clinical features and outcome of infectious endocarditis and vertebral osteomyelitis co-infection. Am J Med. 2014;127(7):e9-e15. doi:10.1016/j.amjmed.2014.02.023 24608019

[23] Stangenberg M Mende KC Mohme M , et al. Influence of microbiological diagnosis on the clinical course of spondylodiscitis. Infection. 2021;49(5):1017-1027. doi:10.1007/s15010-021-01642-5 34254283 PMC8476479

[24] Herren C Jung N Pishnamaz M Breuninger M Siewe J Sobottke R . Spondylodiscitis: diagnosis and treatment options. Dtsch Arztebl Int. 2017;114(51-52):875-882. doi:10.3238/arztebl.2017.0875 29321098 PMC5769318

[25] Gordon RJ Lowy FD . Bacterial infections in drug users. N Engl J Med. 2005;353(18):1945-1954. doi:10.1056/NEJMra042823 16267325

[26] McCann T Elabd H Blatt SP Brandt DM . Intravenous drug use: a significant risk factor for serratia bacteremia. Ther Adv Infect Dis. 2022;9:20499361221078116. doi:10.1177/20499361221078116 35222998 PMC8864268

[27] Rehman S Arif S Ushakumari LG , et al. Assessment of bacterial infections and antibiotic regimens in intravenous drug users. Cureus. 2023;15(9):e45716. doi:10.7759/cureus.45716 37868523 PMC10590200

[28] Wiesseman GJ Wood VE Kroll LL Linda L . Pseudomonas vertebral osteomyelitis in heroin addicts. Report of five cases. J Bone Joint Surg Am. 1973;55(7):1416-1424. 4202276

[29] Levin MH Weinstein RA Nathan C Selander RK Ochman H Kabins SA . Association of infection caused by Pseudomonas aeruginosa serotype O11 with intravenous abuse of pentazocine mixed with tripelennamine. J Clin Microbiol. 1984;20(4):758-762. doi:10.1128/jcm.20.4.758-762.1984 6436316 PMC271426

[30] Heuer A Strahl A Viezens L Koepke LG Stangenberg M Dreimann M . The Hamburg spondylodiscitis assessment score (HSAS) for immediate evaluation of mortality risk on hospital admission. J Clin Med. 2022;11(3):660. doi:10.3390/jcm11030660 35160110 PMC8836753

