# Supplementary material for: Clinical Outcomes and Challenges in the Management of Spondylodiscitis in Patients With Intravenous Drug Abuse: A Multicenter Retrospective Study
Source: Global Spine J. 2025 Nov 18;16(5):2192–201. doi: 10.1177/21925682251399092 (PMC12629966; doi:10.1177/21925682251399092)
Supplement: Suppplemental Material - Clinical Outcomes and Challenges in the Management of Spondylodiscitis in Patients With Intravenous Drug Abuse: A Multicenter Retrospective Study [file sj-pdf-1-gsj-10.1177_21925682251399092.pdf]

**Parameter**	**Non-IVDU (n = 542)**			**IVDU (n = 33)**		
	**Conservative (n = 20)**	**Operative (n = 522)**	**p**	**Conservative (n = 1)**	**Operative (n = 32)**	**p**
**Motor deficit at admission**	20%	35.8%	0.224	0%	37.5%	>0.999
**Motor deficit at discharge**	5%	31.2%	**0.011**	0%	25%	>0.999
**MRC at admission (median, range)**	5 (0-5)	5 (0-5)		5	5 (0-5)	
**MRC at discharge (median, range)**	5 (1-5)	5 (0-5)		5	5 (0-5)	
**Bladder / bowel dysfunction at admission**	0%	14.2%	0.093	100%	9.4%	0.121
**Bladder / bowel dysfunction at discharge**	0%	14.4%	0.093	0%	6.3%	>0.999
**Sepsis at admission**	45%	20.3%	**0.021**	0%	15.6%	>0.999
